# Tactile Object Familiarity in the Blind Brain Reveals the Supramodal Perceptual-Mnemonic Nature of the Perirhinal Cortex

**DOI:** 10.3389/fnhum.2016.00092

**Published:** 2016-04-12

**Authors:** Laura Cacciamani, Lora T. Likova

**Affiliations:** The Smith-Kettlewell Eye Research Institute, San FranciscoCA, USA

**Keywords:** blindness, familiarity, perirhinal cortex, tactile memory, tactile perception

## Abstract

This study is the first to investigate the neural underpinnings of tactile object familiarity in the blind during both perception and memory. In the sighted, the perirhinal cortex (PRC) has been implicated in the assessment of *visual* object familiarity—a crucial everyday task—as evidenced by reduced activation when an object becomes familiar. Here, to examine the PRC’s role in *tactile* object familiarity in the absence of vision, we trained blind participants on a unique memory-guided drawing technique and measured brain activity while they perceptually explored raised-line drawings, drew them from tactile memory, and scribbled (control). Functional magnetic resonance imaging (fMRI) before and after a week of training revealed a significant decrease in PRC activation from pre- to post-training (i.e., from unfamiliar to familiar) during perceptual exploration as well as memory-guided drawing, but not scribbling. This familiarity-based reduction is the first evidence that the PRC represents tactile object familiarity in the blind. Furthermore, the finding of this effect during *both tactile perception* and *tactile memory* provides the critical link in establishing the PRC as a structure whose representations are *supramodal* for *both* perception and memory.

## Introduction

The ability to use the sense of vision to perceive objects in the environment seems instantaneous and effortless and therefore is often taken for granted. Without vision, though, perceiving even the simplest of objects suddenly becomes challenging. Individuals who are blind must rely on other senses, such as their sense of touch, in order to serially explore and understand the world around them. One task that is particularly important to any daily environmental interaction is the ability to assess whether a confronted object is familiar such that an appropriate response to that object can be formed. By “familiar,” we mean that the object has been encountered previously such that stored meaningful object memories exist and can be accessed, thereby making the object recognizable. While a wealth of previous work has investigated this ability to assess object familiarity in the sighted, an understanding of the neural underpinnings of tactile object familiarity in the blind is lacking. The current study provides the first investigation of tactile object familiarity representations in the blind brain.

A key neural structure known to be involved in object familiarity—at least in the visual domain—is the perirhinal cortex (PRC), which lies near the hippocampus in the medial temporal lobe (MTL). The PRC traditionally has been implicated in *memory*—particularly in the recognition of familiar objects (Brown and Aggleton, 2001). Its involvement in recognition memory is typically evidenced not by an increase but by a *reduction* in neuronal responses to visual stimuli that have been encountered previously, thereby rendering them familiar. This familiarity-based response reduction has been observed in single neurons via electrophysiological recordings in monkeys and rats (Ringo, 1996; Brown and Xiang, 1998; Suzuki and Eichenbaum, 2000) as well as via blood oxygen level dependent (BOLD) fMRI in human neuroimaging studies (Henson et al., 2003). Further support for the PRC’s role in visual object familiarity comes from studies showing that damage to or surgical removal of the PRC in monkeys, rats, and humans impairs recognition memory for individual objects (Zola-Morgan et al., 1989; Meunier et al., 1993; Suzuki et al., 1993; Mumby and Pinel, 1994; Buffalo et al., 1998). Moreover, assessing object familiarity seems to be a role specifically associated with the PRC rather than other MTL structures. The hippocampus, for instance, represents the relative spatial locations of objects rather than their relative familiarity (Brown and Aggleton, 2001). The entorhinal cortex (ERC), which others have posited might operate as a bridge between the hippocampus and PRC, signals both object familiarity and spatial location, however, not as strongly as either the PRC or the hippocampus, respectively. Indeed, lesions to the ERC have less severe effects on object recognition abilities than lesions to the PRC (Meunier et al., 1993).

The increased interest in the PRC has led to an abundance of recent work revealing that the PRC’s familiarity representations transcend visual recognition memory and also subserve visual perception (Murray and Bussey, 1999; Bussey et al., 2002, 2005; Murray et al., 2007; Baxter, 2009; Peterson et al., 2012; Nadel and Peterson, 2013). For instance, research in rats, monkeys, and humans has shown that the PRC is involved in the visual discrimination of simultaneously presented complex objects when working memory demands are low (Bussey et al., 2002; Lee et al., 2005; Barense et al., 2007; Bartko et al., 2007). This type of simultaneous object discrimination task has also been used to demonstrate the PRC’s role in perceptual tasks that require access to familiarity representations in particular. For example, when rats are simultaneously presented with two objects—one familiar and one novel—and are allowed to freely explore them, the typical finding is a preference for exploring the novel item—a result indicating the animal’s ability to visually assess and distinguish object familiarity (Ennaceur and Delacour, 1988). Selective damage to the PRC via lesions or chemical antagonists has been shown to impair this discrimination ability such that the rat no longer exhibits a novelty preference, and rather, explores the familiar and novel item equally (Ennaceur et al., 1996; Ennaceur and Aggleton, 1997; Abe et al., 2004). This important work has shown that the PRC is necessary in the visual discrimination of item familiarity.

From the above research, the key involvement of the PRC in visual object familiarity during both perceptual and memory tasks is apparent, thus indicating its *perceptual-mnemonic* functioning. While this previous work has been conducted in the visual modality, other prior research suggests that the PRC’s representations are not unimodal. Given its position at the culmination of the occipitotemporal object-processing stream, the PRC receives input from all sensory modalities (Suzuki and Amaral, 1994), thereby implicating it as a site suitable for cross-modal integration (Murray and Richmond, 2001; Taylor et al., 2006). Indeed, individual neurons in the PRC have been shown to respond to multimodal (both visual and auditory) input (Desimone and Gross, 1979). Neuroimaging studies in humans have shown that the PRC’s representations are stronger during an object-matching task when the input is cross-modal (visual-tactile) vs. unimodal (visual–visual or tactile–tactile) (Holdstock et al., 2009). Furthermore, damage to the PRC results in impaired auditory familiarity assessment during recognition memory tests in sighted humans (Martin et al., 2011). Similarly, in sighted primates, selective lesions to the PRC impair not only visual but also tactile recognition memory (Suzuki et al., 1993), further suggesting that the PRC’s *mnemonic* representations are multimodal, at least in the sighted. Note, however, that these tactile impairments arising from PRC lesions were found *only* for *memory* tasks, while tactile *perceptual* discrimination was unaffected (Suzuki et al., 1993)—an important distinction considering the PRC’s involvement in not only memory but also perception in the visual modality. Thus, it remains unclear whether the ‘dual’ – perceptual and mnemonic – nature of familiarity assessment in the PRC extends beyond the visual modality, and in particular, to the tactile modality. Furthermore, these studies investigated the cross-modal nature of the PRC only in sighted participants. No prior studies have investigated tactile object familiarity in the PRC in blind individuals, including those who have never been able to use vision to form object representations.

The current study aims to fill these gaps in the literature by assessing tactile representations of object familiarity in the PRC in *blind* individuals. Moreover, we aim to determine for the first time whether the PRC’s tactile familiarity representations in the blind are involved in just *memory* or also *perception*. Doing so will provide the missing link in establishing the PRC as not just a *perceptual-mnemonic* structure, but also one that functions independently of sensory modality and visual experience. Furthermore, this study will provide the field’s first glimpse into tactile familiarity in the blind brain—an important contribution that will lay the foundation necessary for future tactile rehabilitation research.

**FIGURE 1:**
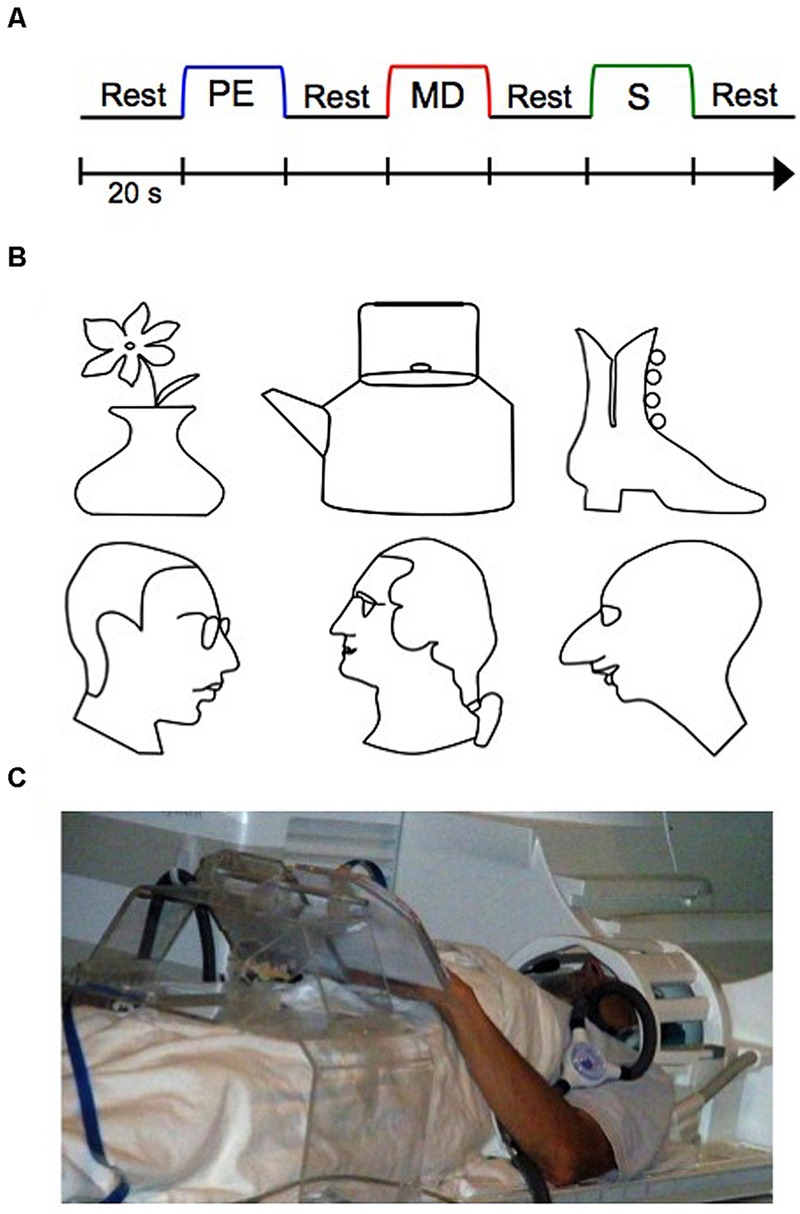
**The experimental functional magnetic resonance imaging (fMRI) design, stimuli, and setup. (A)** Three-task blocked experimental design, repeated twelve times (twice per stimulus). Auditory cues signaled the beginning of each block. **(B)** Raised line-drawing stimuli. **(C)** A participant operating our multimodal MRI-compatible drawing device. PE, Perceptual Exploration; MD, Memory Drawing; S, Scribble.

While no previous work has investigated the blind PRC in particular, evidence of cortical reorganization of the blind brain toward representing tactile information in other brain areas traditionally involved in vision does exist (Amedi et al., 2002; Pascual-Leone et al., 2005; Sathian and Stilla, 2010; Likova, 2010, 2012, 2013). Likova (2010, 2012, 2013) for instance, conducted various studies examining training-based reorganization in the primary “visual” cortex (V1) in the blind as a function of learning. In these studies, blind participants underwent a special training on a demanding tactile memory paradigm (**Figure 1A**), which we employ in the current study as well. In this paradigm, participants learned how to memorize with high precision raised-line drawings (**Figure 1B**) while exploring them one at a time with their left hand (Perceptual Exploration, or PE, condition), and how to use that memory representation for drawing the stimulus from memory using their right hand (Memory Drawing, or MD, condition). As a motor control task, participants also drew randomly with their right hand (Scribble, or S, condition). The use of different hands for exploration and drawing required participants to rely on tactile memory rather than motor representations, rendering this non-visual drawing task even more difficult. Over 5 days, participants were trained on this task, such that by the end of training, participants’ drawings improved substantially. Before and after training, whole-brain fMRI scans were conducted in order to examine the effect of training on cortical reorganization (**Figure 1C**). Likova (2013, 2014) found that only a week of this “Cognitive-Kinesthetic Training” resulted in dramatic changes in the primary visual area V1, consistent across different categories of visual deprivation—congenitally blind, late-onset blind, and blindfolded. BOLD response waveforms in V1 went from being immature and erratic to well-formed and closely fit to the BOLD predictor for the memory-guided drawing task (Likova, 2012). This pre- to post-training difference in activation was not observed in a control area, the motor cortex (M1). This remarkable finding demonstrates that areas typically involved in vision can reorganize to represent tactile memory information in the blind after only 1 week of well-targeted training. The results implicated V1 as the implementation of the theoretical ‘visuo-spatial sketchpad’ for working memory in humans and led to its reconceptualization into a ‘modality-independent (or supramodal) spatial sketchpad’ (Likova, 2012, 2013). Furthermore, this drawing training paradigm has the dual-advantage of being both a *non-visual* and a *causal* intervention. Thus, it made it possible to reveal for the first time complex patterns of training-based *encoding/retrieval* reorganization in the inferotemporal cortex and the hippocampus (Likova, 2015), which has important implications for the emerging view of perception/memory interactions and their dynamics through the learning process.

In the present study, we employed Likova’s Cognitive-Kinesthetic Training (Likova, 2010, 2012, 2013, 2014) to assess perceptual-mnemonic representations of tactile object familiarity in the blind PRC. Participants were trained 2 hours per day for 5 days on how to perceptually explore (PE) the raised-line drawings and subsequently draw them from tactile memory (MD). Although the line drawings depicted well-known, real-world objects, they were unrecognizable, meaningless, and therefore “unfamiliar” to the blind participants before training. After learning these line drawings as a result of the training, object memories were formed such that the line drawings became recognizable, meaningful, and therefore “familiar” to our blind participants after training. Using BOLD fMRI, we compared PRC activation before training (when the objects were unfamiliar) vs. after training (when the objects were familiar) during both the tactile perception and memory tasks (PE and MD). We hypothesized that, if the PRC does indeed represent tactile object familiarity in the blind as it does for visual object familiarity in the sighted, then we should observe the PRC’s signature pattern of recognition familiarity—that is, a *decrease* in BOLD activation when the items become familiar (i.e., from pre-training to post-training). To ascertain whether this effect is specific to the PRC (as hypothesized) or is a more general effect that spreads to or leaks from other nearby MTL regions, we also assessed pre- to post-training differences in activation in the ERC, which is the MTL region most proximal to the PRC.

## Materials and Methods

### Participants

The participants were eight blind individuals (four females, four males; ages 31–76) whose demographics are summarized in **Table 1**. All participants gave informed consent for the experimental protocol, which was approved by the Smith-Kettlewell Institutional Review Board, and were compensated for their time. All participants were right-handed.

**Table 1:** Participant demographics.

participant #	Gender	Age	Current visual status	Age of onset of current visual status	Visual status at birth	Did participant ever have full vision?	Could participant ever use vision to see shapes/objects?	Diagnosis	Braille fluency
1	M	68	NLP	15	LP	**No**	**No**	Retinopathy of prematurity	High
2	F	66	LP	<1	LP	**No**	**No**	Retinopathy of prematurity	High
3	F	57	LP	30	Tunnel vision	**No**	Yes	Retinitis pigmentosa	High
4	M	76	LP	16	Full vision	Yes	Yes	Optic neuropathy	Moderate
5	F	31	NLP	28	LP	**No**	Yes	Optic nerve hypoplasia	High
6	F	66	NLP	16	Full vision	Yes	Yes	Glaucoma	Moderate
7	M	70	LP	60	Full vision	Yes	Yes	Optic neuropathy	None
8	M	56	LP	47	Full vision	Yes	Yes	Glaucoma	None

*NLP, no light perception; LP, light perception.*

To ascertain the current visual status of all participants, the Berkeley Rudimentary Vision Test (Bailey et al., 2012) was administered during which a series of cards with black and white tumbling E’s, gratings, and field projections were presented. None of the participants reported being able to perceive any of the information on these cards with either eye. Light perception was then assessed via a flashlight. Five participants had some light perception (LP) in segments of the visual field in one or in both eyes; for these participants, a blindfold was placed over their eyes for the duration of the experiment to eliminate all possible visual input. The other three participants were unable to perceive this light, thereby being classified as having no light perception (NLP). Of these NLP participants, two (#1 and #2) were totally blind (LP) from birth and therefore were never able to use vision to form any object representations. While our blind participants did differ with respect to their age of blindness onset, that we included these congenitally blind participants allows us to speak to whether vision is necessary in forming tactile familiarity representations in the PRC.

### Design

The experimental design was as in Likova (2012, 2013, 2014; see **Figure 1**). In short, the key component of the study was applying the Cognitive-Kinesthetic Drawing Training as a powerful memory intervention instrument that allowed us to achieve remarkable *causal* changes at a behavioral level within only 5 days of 2 h/day sessions. Before and after completing the training, the participants were tested by fMRI. In the scanner, participants performed three tactile tasks in a block paradigm (see **Figure 1A**) as in Likova (2012, 2013, 2014). The three tasks were as follows: during *Perceptual Exploration* (PE), participants explored a raised line drawing with their left hand (a task that is predominantly perceptual, though—as any perception—it does involve memory encoding); during *Memory Drawing* (MD), participants used their tactile memory to draw the same image with their right hand (a non-perceptual task that requires not only access to but also implementation of the memory); and during *Scribble* (S), participants drew randomly with their right hand as a motor control (no perception or access to memories is needed). Each task lasted 20 s, and participants were instructed to continue drawing/exploring for the entire 20 s, even if they finished early, so that equal time was spent on each task. After 20 s, the participants were told to stop what they were doing regardless of their progress. The tasks were separated by a 20-s rest interval (RI) during which participants were instructed to clear their mind of any shapes or images. An auditory cue signaled the start of each task. The three-task sequence (RI, PE, RI, MD, RI, S, RI) was repeated twice for each line-drawing stimulus, and there were six stimuli (three faces and three objects; see **Figure 1B**), for a total of 12 repetitions of the sequence. Prior to beginning the pre-training fMRI session, participants were informed as to the nature of the experiment and briefly familiarized with the tasks and equipment.

During the training sessions, participants were trained on how to efficiently and accurately perform the three tasks. At the start of training, participants were allowed to explore and draw the objects without the 20 s time restriction while learning the detailed spatial components of the line drawings. As training progressed, the 20 s time limit for each task was enforced. Importantly, the same line drawings were used during training and during fMRI scanning (see **Figure 1B**); thus, participants became highly familiar with the set of stimuli.

### Equipment

A unique custom MRI-compatible presentation and drawing system was used for the brain imaging portion of this study (see **Figure 1C**). The system consisted of a plexiglass table extending across the participant’s lap with a dual-slot adjustable surface attached on the top. The left slot held the line drawing stimulus, while the right slot held an MRI-compatible electronic drawing tablet. Movement of the stylus (held in the participant’s right hand) across this drawing tablet was recorded and presented in real-time on the control computer such that the participant’s drawings were viewable to the experimenters. The auditory stimuli were presented through Resonance Technologies earphones (Resonance Technologies, Salem, MA).

### fMRI Acquisition and Analyses

Data were collected on a Siemens Trio 3T magnet equipped with a 12-channel head coil. BOLD responses were obtained using an EPI acquisition (TR = 2 s, TE = 28 ms, flip angle = 80^o^, voxel size = 3.0 mm × 3.0 mm × 3.5 mm) consisting of 35 axial slices extending across the whole brain. Pre-processing was conducted using FSL (Analysis Group, fMRIB, Oxford, UK) and included slice-time correction and two-phase motion correction, consisting of both within-scan and between-scan 6-parameter rigid-body corrections. To facilitate segmentation and registration, a whole-brain high-resolution T1-weighted anatomical scan was also obtained for each participant (voxel size = 0.8 mm × 0.8 mm× 0.8 mm). White matter segmentation in this T1 scan was conducted using FreeSurfer (Martinos Center for Biomedical Imaging, Massachusetts General Hospital) and gray matter was generated with the mrGray function in the mrVista software package (Stanford Vision and Imaging Science and Technology).

In order to obtain estimates of neural activation amplitudes for each task, a general linear model (GLM) was fit to the acquired BOLD data for each three-task sequence. The model consists of three boxcars representing the task activations plus a sequence of impulses corresponding to auditory cues convolved with an estimated hemodynamic response function (HRF), and a fourth-order polynomial for low-frequency baseline fluctuations. For each task (PE, MD, and S), statistical parametric maps (SPMs) were generated based on the estimated activation amplitudes from the above GLM in each voxel that exceeded the noise threshold defined by the variability in the residual. Voxel-wise difference *z*-score maps were also created in order to compare pre-training and post-training activation.

### Region of Interest Analysis

The main region of interest (ROI) in the present study was the PRC. The left and right PRC ROIs were defined anatomically in each participant based on previously determined guidelines (Insausti et al., 1998; Kivisaari et al., 2013). Although there are often substantial individual differences in MTL anatomy, care was taken to define ROIs as consistently as possible between participants. Specifically, from anterior to posterior, the PRC was defined as beginning 2 mm anterior to the appearance of the *limen insulae* gray and ending 3 mm posterior to the disappearance of the ERC (see next paragraph). From medial to lateral, the PRC extended from the shoulder of the medial bank of the collateral sulcus (CS) to the shoulder of the lateral bank of the CS.

In order to ascertain that any training-induced effects we observed were specific to the PRC and not a general MTL effect, we also created left and right ROIs for the ERC. This structure, like the PRC, was defined based on pre-determined guidelines (Kivisaari et al., 2013). From anterior to posterior, the ERC was defined as beginning 2 mm posterior to the first appearance of the *limen insulae* white and ending 1 mm posterior to the apex of the intralimbic sulcus. From medial to lateral, the ERC extended from the medial apex of the parahippocampal gyrus to the shoulder of the medial border of the CS (i.e., up to but not extending into the CS).

**FIGURE 2:**
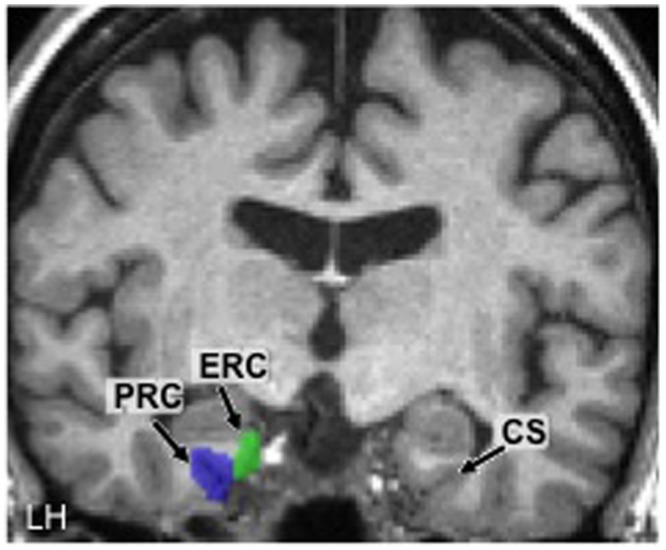
**Hand-drawn medial temporal lobe regions of interest (ROIs) in the left hemisphere (LH) in one participant.** The collateral sulcus (CS) is shown in the other hemisphere as a point of reference. PRC, perirhinal cortex; ERC, entorhinal cortex.

Using these anatomical landmarks, ROIs for the left and right PRC and ERC were hand-drawn in each individual brain in FSL (Smith et al., 2004). **Figure 2** shows an example of these ROIs in the left hemisphere in one representative participant (#2).

The Stanford package mrVista was used to estimate the neural activation amplitudes for each task within these PRC ROIs using the same GLM procedure as above, but applied to the average signal across all voxels within the ROI.

## Results

### Participant Report

As expected, participants reported that the tactile memory-guided drawing task was quite challenging. Prior to training, some participants were not even familiar with how to properly hold a pen or with simple spatial attributes present in the raised-line drawings, such as curved vs. straight lines. When participants explored the stimuli with their left hand during PE, they initially reported (in between scans during the pre-training fMRI session) being unable to recognize the line drawings or clearly understand their detailed spatial components. When trying to draw the raised-line images from tactile memory with their right hand during MD, participants lacked confidence and often expressed conviction in the impossibility of such a task. Importantly, the use of the left hand for exploration and the right hand for drawing ensured that participants could not rely on motor memory or haptic knowledge to produce their drawings; instead, they had to rely on their memory representation of the stimulus. Moreover, by separating their two hands, participants could not employ the common strategy of using their left hand to guide the pen as they drew with their right hand, thereby making the drawing task even more challenging, as this two-hand technique is often used by blind individuals during Braille reading and writing.

After the Cognitive-Kinesthetic training, however, participants could easily recognize each stimulus during exploration, and after a 20 s rest period, could use their memory representation of the stimulus to confidently create a drawing (in only 20 s) that closely resembled the original stimulus by ‘projecting’ it onto the drawing space on the right side of the device. Participants reported being more aware of not only the identity of each stimulus, but also the detailed spatial components that comprised it. On a more humanistic level, participants expressed certainty, happiness, and excitement with their drawings after training—a massive shift in mental and emotional state from before training, with respect to not only the task but also their self-confidence. Together, these self-reports imply that the Cognitive-Kinesthetic training allowed participants to create robust memory representations of the line-drawing stimuli, thereby rendering them familiar.

### Drawing Results

**FIGURE 3:**
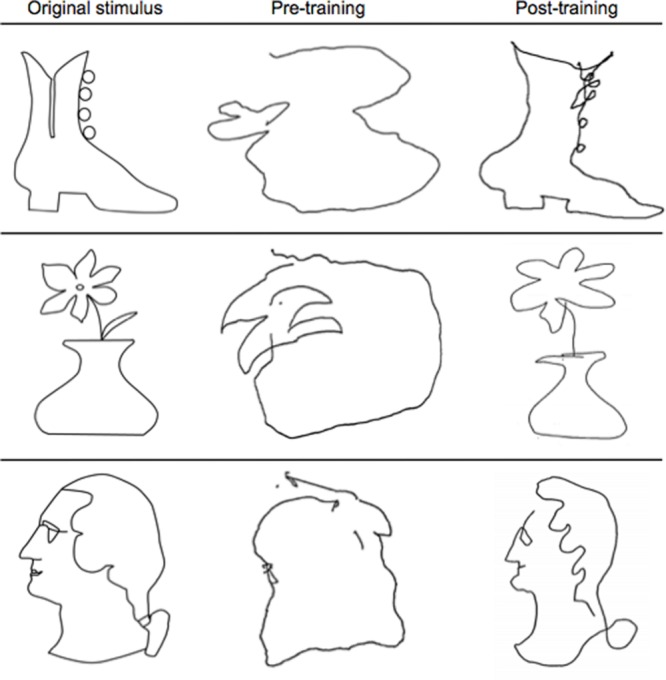
**Representative examples of pre- and post-training drawings.** Note the substantial improvement from pre-training (middle column) to post-training (last column) in resembling the original stimulus (first column) after just 5 days of the Cognitive-Kinesthetic training.

Participants’ memory-guided drawing ability improved substantially from pre- to post-training (see **Figure 3**). Drawing speed was radically enhanced, from a median value of 3 min per drawing at the beginning of training (when time was not restricted) to typical achievement of the complete drawing in the target time of 20 s or less at the end of training. Before training, drawings were disconnected, unstructured, and most often unrecognizable. After training, the drawings clearly resembled the original stimuli, consistent with participants’ reports of being able to recognize the stimuli and create strong mental representations of their spatial arrangements. Individual differences were apparent in the participants’ drawing abilities, as would be expected; even so, every participant’s drawings improved significantly as a result of the training, even in spite of the physical limitations of the narrow bore of the scanner.

### fMRI Results

#### Perirhinal Cortex

##### ROI analysis

**FIGURE 4:**
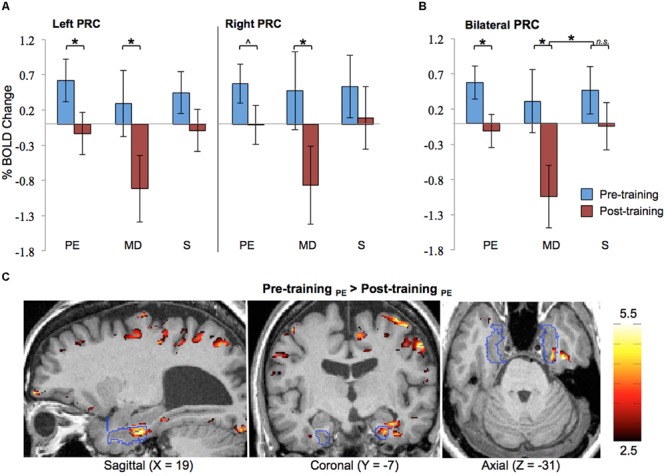
**Functional magnetic resonance imaging results.** ROI analysis results for pre- and post-training are shown **(A)** separated by hemisphere and **(B)** collapsed across hemisphere. Error bars represented standard error of the mean of the difference scores (post-pre). **(C)** Pre-training > post-training difference *z*-score map in one participant (#2), with the PRC ROI outlined in blue. PRC, perirhinal cortex; PE, Perceptual Exploration; MD, Memory Drawing; S, Scribble; ^∗^*p* < 0.05, ˆ*p* < 0.10.

Results of the ROI analysis on BOLD activation in the left and right PRC are shown in **Figure 4A**. The data shown here represent the averages across all eight blind participants. The results show that prior to Cognitive-Kinesthetic drawing training (i.e., when the stimuli were unfamiliar), activation in the left and right PRC was significantly above zero (*p*s = 0.04 and 0.03, respectively) during exploration of the raised-line drawing (PE task). After training, when the stimuli were familiar, PRC activation during PE was reduced to baseline. This pre- to post-training decrease was significant in the left PRC (*p* = 0.04) and marginally significant in the right PRC (*p* = 0.07). Indeed, there was no main effect of hemisphere (*p* > 0.40), indicating that the pattern of activation did not significantly differ in the left vs. right PRC. For this reason, we collapsed the data across hemispheres (see **Figure 4B**). An analysis on these cross-hemisphere data confirms a significant decrease in PRC activation from pre- to post-training during the PE task (*p* = 0.02), with pre-training activation significantly above baseline (*p* = 0.008) and post-training activation not significantly different from baseline (*p* > 0.70).

PRC activation during the MD task was much more variable both within and between participants, as can be expected for this more complex learning task of memory-guided drawing. Specifically, during MD, participants must access a detailed memory representation of the stimulus while recruiting precise motor control to produce a drawing containing detailed spatial components. Despite all of these intricacies and the resulting high variability, a significant reduction in activation from pre- to post-training was observed in both the left and right PRC (*p*s = 0.04 and 0.04, respectively) during the MD task (see **Figure 4A**). The BOLD responses shown here represent signals averaged across both space (all voxels within the ROI) and time (the 20-s task interval). The data collapsed across hemispheres continue to show this significant pre- to post-training decrease in activation (see **Figure 4B**; *p* = 0.01), with post-training activation decreasing significantly below baseline (*p* = 0.004). Moreover, this pre- to post-training reduction during the MD task was significantly greater than the pre- to post-training difference during the S control task, as evidenced by a significant session × task interaction (*p* = 0.03).

As expected, no significant differences in activation between pre- and post-training were observed during the control task (S) in either the left or right PRC.

The main finding from the above ROI analysis is a *reduction* in PRC activation from pre- to post-training during perceptual exploration (PE task) and memory-guided drawing (MD task) of the line drawing stimuli across all participants, consistent with their becoming familiar with the stimuli by the repeated exposure during the training period.

##### Cluster analysis

To ascertain the robustness of this familiarity-based reduction in PRC activation in each individual participant, we also generated difference *z*-score maps comparing pre- vs. post-training to perform a cluster analysis. In every participant, a cluster of significant voxel activation differences (*z* > 1.96, *p* < 0.05 per voxel) was found in both the left and right PRC during the PE task in the direction of pre-training > post-training (see **Table 2**; **Figure 4C** depicts this significant cluster in the right hemisphere in one representative participant). This significant familiarity-based reduction in activation from pre- to post-training during PE in each participant is consistent with the above analysis collapsed across participants. That the effect is observable both within and across participants speaks to its robustness, despite the minor variations in location of the maximum familiarity-based reduction.

**Table 2:** Clusters in the PRC showing significant pre-training > post-training differences.

Participant #	Left		Right	
	Max Z	Peak coordinates	Max Z	Peak coordinates
**PE task**				
1	2.25	-31, -8, -25	3.01	33, -13, -36
2	3.84	-28, -14, -22	5.6	21, -16, -27
3	3.31	-27, -8, -25	2.44	32, 6, -31
4	6.87	-31, -20, -23	5.52	35, -24, -29
5	3.15	-27, -6, -26	2.91	21, -27, -17
6	6.22	-30, -10, -23	4.7	25, -9, -22
7	2.40	-30, -6, -26	2.12	40, -18, -22
8	8.33	-33, -11, -33	8.91	33, -9, -33
**MD task**				
1	2.76	-31,-16,-22	-	-
2	3.5	-28, -13, -26	3.2	23, -14, -25
3	4.69	-27, -8,-26	5.23	30, -10, -27
4	5.39	-32, -18, -24	2.28	26, -10, -26
5	3.05	-36, -25, -20	3.08	33, -22, -26
6	3.56	-30, -8, -24	7.17	26, -6, -26
7	2.63	-36, -15, -29	3.65	36, -9, -26
8	8.53	-33, -12, -32	9.41	34, -11, -22

*PE, Perceptual Exploration, MD, Memory Drawing; coordinates are in Talairach space (x, y, z).*

The cluster analysis also revealed a suprathreshold cluster of significant voxel activation differences (*z* > 1.96, *p* < 0.05 per voxel) in the direction of pre-training > post-training during the MD task in the left PRC in every participant and in the right PRC for seven out of the eight participants. This result is consistent with the significance observed in the ROI analysis across participants during the MD task.

Together, these results indicate that the familiarity-based reduction in activation observed in the PRC during PE (a predominately perceptual task) is also apparent during MD (a non-perceptual memory task). This finding in the blind coincides with previous work in the sighted showing that the PRC is involved in familiarity representations that subserve both perception and memory.

#### Entorhinal Cortex

In order to ascertain that our familiarity-based reduction in activation was an effect specific to the PRC, we conducted ROI analyses on the immediately adjacent ERC ROI as well. As in the PRC, there was no main effect of hemisphere in this area (*p*s > 0.30), allowing us to collapse the data across left and right ROIs. However, in contrast to the PRC, analyses on the ERC data produced no significant differences between pre-training and post-training activation in any of the three task conditions (PE, MD, or S), *p*s > 0.20. The lack of a pre- to post-training difference in the ERC could suggest that our tactile familiarity effect is restricted to the PRC, or that the weaker involvement of the ERC in recognition was not sufficient to produce a measureable difference in activation. Future research could elucidate the ERC’s role in tactile object familiarity.

## Discussion

This study is the first to examine the ability of the PRC to represent tactile—rather than visual—object familiarity *in the blind*. Furthermore, it investigates familiarity representations during *both* perception and memory. “Familiarity” in this sense refers to the prior exposure to an object such that the object is meaningful and recognizable to the observer. Previous work in the sighted shows that the signature pattern of PRC activation in representing object familiarity is a *decrease* in neural activity when an item is made experimentally familiar (Ringo, 1996; Brown and Xiang, 1998; Suzuki and Eichenbaum, 2000; Brown and Aggleton, 2001; Henson et al., 2003). This effect has been observed in the visual modality for both memory and perception. While there is some prior evidence of cross-modal representations in the PRC (Desimone and Gross, 1979; Suzuki et al., 1993; Holdstock et al., 2009; Martin et al., 2011), evidence of tactile representations *per se* is limited and is restricted to *memory only*. Furthermore, the PRC’s involvement in tactile memory has previously only been investigated in the sighted. Thus, it remains unclear whether vision is necessary in establishing familiarity representations in the PRC, and whether there are supramodal familiarity representations for not memory only but perception as well.

Here, we used a unique fMRI learning paradigm to show that the PRC represents tactile object familiarity in the blind, including those who have never had full vision. Specifically, after 5 days of the Cognitive-Kinesthetic training (Likova, 2010, 2012, 2013, 2014) during which participants became able to perform memory-guided blind drawing and became familiar with a set of raised-line drawings of faces and objects, PRC activation significantly decreased bilaterally in response to increased familiarity with the stimuli. This familiarity-based reduction was observed in the group analysis as well as the individual cluster analysis, thereby speaking to the robustness of this pre- to post-training decline. Thus, the results from this study show for the first time that the PRC represents tactile object familiarity in the blind, and that these representations can form independently from visual experience. By revealing that the PRC can reorganize to represent tactile information when necessary, this work has important implications for the fields of perception and memory as well as for the field of blindness rehabilitation.

The familiarity-based reduction in PRC activation in the present study was observed during PE when participants were exploring the raised-line drawings (a task that is predominantly perceptual, but does involve access to and encoding of object memories) and during MD when they were drawing the images from tactile memory (a non-perceptual memory retrieval task). This finding is consistent with recent work in the visual modality which has shown that the PRC’s familiarity representations subserve both memory and perception (Murray and Bussey, 1999; Bussey et al., 2002; Murray et al., 2007; Baxter, 2009; Peterson et al., 2012; Nadel and Peterson, 2013). For instance, the PRC has been implicated in visual recognition memory (Ringo, 1996; Brown and Xiang, 1998; Suzuki and Eichenbaum, 2000; Brown and Aggleton, 2001; Henson et al., 2003) and also in visual familiarity/novelty discrimination when two objects are presented simultaneously (Ennaceur et al., 1996; Ennaceur and Aggleton, 1997; Abe et al., 2004). During such perceptual discriminations, existing stored familiarity representations must be accessed and matched to the currently perceived visual input. Likewise, in the current study, when participants tactually explore the raised-line drawings during PE, they are attempting to match the perceived stimulus to an existing familiarity representation—in this case, one created via the tactile modality. Similarly, during MD, the existing familiarity representations must be accessed in detail in order to guide drawing. In this way, the familiarity representations created during the Cognitive-Kinesthetic training are used to facilitate both PE and MD. As such, both of these tasks rely on access to memories; the difference lies in the reason for access and how that memory is utilized.

Overall, the results of the present tactile perception and memory study show for the first time that the PRC is not only *perceptual-mnemonic* in nature—as in the visual modality—but moreover, since these familiarity effects were found in the tactile modality, that the PRC’s *perceptual* and *mnemonic* representations are *supramodal*.

The finding of supramodality in the PRC in the present study adds to previous work revealing the supramodal nature of other cortical areas, as assessed in blind individuals. For instance, Likova (2010, 2012, 2013) has shown that low-level “visual” regions, including V1 and extrastriate cortex, can reorganize to represent tactile information after just 5 days of the Cognitive-Kinesthetic training. Even without this training, the blind visual cortex is active during certain tactile and auditory tasks, including reading, discriminating Braille (Sadato et al., 1996, 2002) and hearing echoes produced during echolocation (Thaler et al., 2011). Higher in the representational hierarchy, the inferotemporal cortex and even the hippocampus have been shown to undergo training-induced reorganization toward tactile representations in the blind as well (Likova, 2015). Additionally, areas of the parietal cortex typically implicated in visual spatial attention—including the intraparietal sulcus and superior parietal lobule—have also been shown to be supramodal in nature (Leo et al., 2012; Ricciardi et al., 2014). Moreover, these parietal areas have strong functional connectivity with the visual cortex during tactile perception (Leo et al., 2012), further pointing toward the supramodality of not only individual cortical regions but also the networks in which they are involved. The current study goes further to demonstrate that such a key MTL region as the PRC is also a supramodal structure, with its object familiarity representations apparent in the tactile domain. This work, along with the previous literature, indicates that visual experience is not necessary in forming spatial representations.

It is interesting to consider whether the familiarity-based decrease in PRC activation observed in the present study is due to familiarity at the perceptual or semantic level. Previous research has shown that the PRC indeed represents high-level semantic information pertaining to objects (Bruffaerts et al., 2013; Clarke and Tyler, 2014) such that the assessment of an object’s meaningfulness is impaired when the PRC is compromised, as in semantic dementia (Barense et al., 2010b). Our definition of “familiarity” in the present study is very closely related to (if not synonymous with) “meaningfulness”; thus, our observed familiarity effects are likely at the level of semantic familiarity. That we found our familiarity effect during the MD task where participants must rely on mental representations rather than immediate perceptions supports this semantic-level familiarity. Of course, participants did develop a familiarity with both perceptual and semantic components of the stimuli over the course of the training, and given the PRC’s involvement in both perception and memory (e.g., Murray and Bussey, 1999; Bussey et al., 2002, 2005; Murray et al., 2007; Baxter, 2009; Peterson et al., 2012; Nadel and Peterson, 2013), it likely represents familiarity at multiple levels.

Previous research on the PRC (in the visual modality) has not only demonstrated its involvement in representing object familiarity, but also in representing objects comprised of complex conjunctions of features (Bussey et al., 2005). For instance, neurophysiological research has demonstrated that monkeys and rats with selective lesions to the PRC are impaired on visual discrimination tasks when the objects possess a high degree of feature ambiguity—that is, when the objects have multiple features in common (Bussey and Saksida, 2002; Bussey et al., 2002; Bartko et al., 2007). Similarly, humans with damage to the MTL including the PRC are impaired at object discrimination under conditions of high feature ambiguity, whereas humans with damage restricted to the hippocampus do not exhibit this deficit (Lee et al., 2005; Barense et al., 2007). FMRI studies have provided further supporting evidence for the PRC’s involvement in feature complexity/ambiguity, showing that PRC activity is higher during a discrimination task when the objects possess a high vs. low degree of feature ambiguity, even when controlling for level of difficulty (Barense et al., 2010a). To date, no studies have investigated this feature-conjunction model of PRC function using tactile stimuli or in those who are blind. In the present study, we did not directly manipulate feature complexity or ambiguity of our tactile raised-line drawing stimuli. However, all of our line drawings could be considered complex tactile stimuli, as they depicted whole, real-world objects comprised of many complex features (eyes, hairlines, petals, handles, etc.; see **Figure 1B**). In designing our stimuli this way, we may have increased the probability of PRC involvement in representing each individual stimulus. Likewise, that we found PRC involvement for these complex stimuli support feature conjunction models of PRC function (Bussey and Saksida, 2002; Cowell et al., 2006). Across our set of stimuli, the tactile line drawings did possess some degree of feature ambiguity; for instance, the faces all were comprised of eyes, noses, and chins. Thus, it is possible that the PRC was recruited (during both PE and MD) in order to differentiate between the highly similar and complex stored representations across these stimuli. Separate studies would need to be conducted in order to fully understand how the blind PRC represents tactile objects with differing degrees of feature ambiguity and complexity.

Although our raised-line drawing stimuli did include both faces and objects, we did not have the statistical power needed to conduct an analysis comparing the two categories given our small set of stimuli, number of repetitions, and number of participants. The PRC has been shown to respond to many different categories of complex objects, including faces (Barense et al., 2010a), objects (Ennaceur and Aggleton, 1997; Bussey et al., 2002; Lee et al., 2005; Barense et al., 2007, 2010b; Bartko et al., 2007), words (Bruffaerts et al., 2013), and even meaningless stimuli like blobs and “greebles” (Barense et al., 2007, 2010b). Thus, had we been able to conduct a categorical analysis, we might not have expected to observe a difference between faces and objects in the PRC. Other areas of the inferior temporal cortex, though, have been shown to differentiate between stimulus categories (e.g., Kriegeskorte et al., 2008; Clarke and Tyler, 2014). Future research should elucidate differences in activation between different object categories along the visual hierarchy in the blind.

## Conclusion

The results of the present study show for the first time that the PRC represents tactile object familiarity in humans, and particularly in blind individuals. Furthermore, our finding of object familiarity effects in the PRC in both *tactile perception* and *tactile memory* in blindness provides the critical link to the establishment of the PRC as a *supramodal perceptual-mnemonic* brain structure. Future research could further explore the underlying architectural principles and familiarity-related role of other MTL structures, such as the ERC, in non-visual perception and memory in the blind.

## Author Contributions

LC and LL both contributed to the idea, participant recruitment, implementation, data analysis, and writing of the manuscript. LL developed the experimental design, the Cognitive-Kinesthetic method used to train the blind people to draw, and conducted the training of each participant.

## Conflict of Interest Statement

The authors declare that the research was conducted in the absence of any commercial or financial relationships that could be construed as a potential conflict of interest.
